# Sensorimotor Research Utilising Immersive Virtual Reality: A Pilot Study with Children and Adults with Autism Spectrum Disorders

**DOI:** 10.3390/brainsci10050259

**Published:** 2020-04-29

**Authors:** Irene Valori, Rena Bayramova, Phoebe E. McKenna-Plumley, Teresa Farroni

**Affiliations:** 1 Department of Developmental Psychology and Socialisation, University of Padova, 35131 Padova, Via Venezia 8, Italy; irene.valori.1@phd.unipd.it; 2 Department of General Psychology, University of Padova, 35131 Padova, Via Venezia 8, Italy; renabayramova@outlook.com; 3 School of Psychology, Queen’s University Belfast, Belfast BT9 5BN, UK; pmckennaplumley01@qub.ac.uk

**Keywords:** autism spectrum disorder, ASD, vision, proprioception, self-motion, immersive virtual reality, IVR, HMD, technology

## Abstract

When learning and interacting with the world, people with Autism Spectrum Disorders (ASD) show compromised use of vision and enhanced reliance on body-based information. As this atypical profile is associated with motor and social difficulties, interventions could aim to reduce the potentially isolating reliance on the body and foster the use of visual information. To this end, head-mounted displays (HMDs) have unique features that enable the design of Immersive Virtual Realities (IVR) for manipulating and training sensorimotor processing. The present study assesses feasibility and offers some early insights from a new paradigm for exploring how children and adults with ASD interact with Reality and IVR when vision and proprioception are manipulated. Seven participants (five adults, two children) performed a self-turn task in two environments (Reality and IVR) for each of three sensory conditions (Only Proprioception, Only Vision, Vision + Proprioception) in a purpose-designed testing room and an HMD-simulated environment. The pilot indicates good feasibility of the paradigm. Preliminary data visualisation suggests the importance of considering inter-individual variability. The participants in this study who performed worse with Only Vision and better with Only Proprioception seemed to benefit from the use of IVR. Those who performed better with Only Vision and worse with Only Proprioception seemed to benefit from Reality. Therefore, we invite researchers and clinicians to consider that IVR may facilitate or impair individuals depending on their profiles.

## 1. Introduction

Children with Autism Spectrum Disorders (ASD) can present various types of sensory atypicalities including hypersensitivity, hyposensitivity, and unique patterns of response to sensory stimuli [1], higher reliance on unimodal processing [2], and an extended (hence less precise and specialised) multisensory temporal binding window [3]. These are early symptoms that can be associated with a broad range of cascading delays and impairments [4]. Early motor development might also be affected, as it has been hypothesised that the acquisition of body knowledge develops based on our sensitivity to sensorimotor contingencies (action–consequences correspondence) and multisensory contingencies (correspondence between events in different sensory modalities) [5]. When learning a new movement, there is evidence that children with ASD are less influenced by visual feedback [6] and that they perform better than neurotypical children when the motor learning is driven by proprioceptive input [7]. For instance, the authors asked typically developing children and children with ASD to reach a target by holding a robotic arm. In some random trials, the robotic arm was perturbed and unexpectedly influenced the children’s reaching movement. In the following trial, a learning-from-error effect would lead to an altered movement, which was planned to compensate for the perturbation. The perturbation could be presented to children either through visual feedback (displacement of the cursor representing the robotic arm on the screen) or proprioceptive feedback (a force imposed on the robotic arm). Compared to typically developing children, children with ASD showed a higher sensitivity to when learning from proprioceptive feedback and a lower one when learning from visual feedback [7]. Indeed, motor learning occurs thanks to internal models of action: the association between self-generated motor commands (efferent systems) and sensory feedback from the body and the external world (afferent systems), so that it is possible to predict what would happen as the consequence of an action [6]. Information from muscle, joint, and skin receptors constitute our *proprioception*, the awareness of the position and movement of our body in space which is crucial to the production of coordinated movements [8]. Children with ASD show “an abnormal bias towards reliance on proprioceptive feedback from their own bodies, as opposed to visual feedback from the external world”, which might predict impairments in motor control, social skills, and imitation ability [9] (p. 10). In learning motor sequences, adults with ASD also show deficits in the use of vision, which is the sense that neurotypical adults rely on, but preserved proprioception-driven learning [10]. Neurotypical adults have been found to experience a postural illusion (which manifests as a forward lean) when exposed to an intermittent vibratory stimulation of the posterior side of the neck, as long as vision was occluded. On the other hand, those with ASD experienced the illusion even when vision was available, demonstrating limited contribution of vision in modulating proprioception [11]. While the majority of research supports this over-reliance on proprioception, some research has contrastingly related motor impairments in ASD to an over-reliance on vision and proprioceptive deficits [12,13]. However, these studies utilised small sample sizes and limited data analyses. Meanwhile, neuroimaging research has shown associations between ASD severity and asynchronous functional connectivity between visual and motor networks in children at rest [14], reduced functional connectivity between visual areas and somatosensory motor networks, and increased connectivity between the cerebellum and sensorimotor areas in both children and adults at rest [15]. The remaining question is whether there is a general trend of over-reliance on proprioceptive over visual cues at the root of sensorimotor atypicalities in ASD. If that were the case, early interventions could potentially be aimed at increasing the reliance on vision in children with ASD, moving them away from this proprioceptively dominant processing. Such training should improve their sensorimotor functioning, potentially leading to benefits for cognitive, social, and communicative skills. 

Immersive Virtual Reality (IVR) is particularly appropriate to this end as it allows for controllable input stimuli and the tracking and monitoring of individuals’ actions in a safe learning situation where an individualisation of assessment and training is possible [16]. Moreover, this technology makes it possible to manipulate individual sources of sensory information (e.g., visual, vestibular, or proprioceptive) that are physiologically bound together and induce a mismatch between them to study the role of each sensory modality with respect to accuracy in different tasks [17]. For instance, we can disentangle the contribution of visual and proprioceptive inputs to body perception and movement. In this respect, the most promising IVR tools are head-mounted displays (HMDs), which block out the external world, fully immerse the user in the virtual stimulation, and foster a subjective sense of presence in the virtual world [18]. The result is physiological, emotional, and behavioural responses that are consistent with the physical existence of the virtual world [18]. Despite the broad research and intervention potential offered by HMDs, they have unique features that lead to sensorimotor interactions that do not constitute an exact corollary for real-world experience. Valori and colleagues [19] found that self-motion performance worsened in IVR conditions with vision available relative to the same conditions in reality and indeed, the way that HMDs deliver visual information has essentially unknown effects on movement and its perception [20].

Most notably, the extant literature seems to neglect a developmental point of view, which is only recently being addressed [21]. It seems that technology-driven peculiarities of IVR and HMDs may induce different sensorimotor effects depending on the user’s developmental stage, as has been found in research with neurotypical children and adults. Indeed, when neurotypical people have to learn a walking path while wearing an HMD, adults seem not to benefit from multisensory (visual + self-motion) versus unimodal information, while children of 10−11 years old could benefit from the multisensory learning condition [22]. Therefore, we should investigate the interaction between developmental trajectories of users and the peculiarities of technologies. This would make it possible to understand the unique potentialities and limitations that IVR might have for specific populations with typical or atypical development. At the very beginning of the investigation of the potentialities and limitations related to the use of virtual reality tools for individuals with atypical developmental trajectories and sensory, motor, and cognitive atypicalities, 2D non-immersive systems were preferred due to the technological limits of IVR (graphic quality, limited field of view, temporal lag, size and weight, movement restriction, aftereffects of motion sickness, costs, and accessibility) [23]. Although almost two decades have passed, IVR has greatly improved, and HMDs are sometimes used in research and practice with neurodevelopmental disorders; to our knowledge, only one study has investigated the specific aspects of the interaction between atypical development and the atypicality of interacting with virtual environments. Simões et al. suggest that individuals with ASD may show similar social behaviours (i.e., interpersonal distance) in virtual and real environments, even though neurotypical controls differently interact with a real versus virtual person [24]. We hypothesise that HMDs have unique features that are relevant for people with ASD. This technology seems to intrinsically generate a conflict between vision and proprioception and disrupt the reliability of proprioception [19], potentially reducing its hyper-reliance in ASD. Furthermore, HMDs provide visual information that does not perfectly resemble that of the real world, and they might foster the use of the ventral visual pathway (for object qualities) rather than the dorsal pathway (for movement and spatial aspects of stimuli) [25]. This could suit the visual atypicalities of ASD, which are suggested to present impairments in the dorsal pathway [26], allowing individuals with ASD to interact with the world through the visual mechanisms that are most effective for them. However, several issues should be considered when designing virtual environments for specific purposes in sensorimotor research and interventions for individuals with ASD. Firstly, given that there are usually no binocular cues in IVR, action and perception of depth and motion will be achieved through the ventral stream, which will require much heavier input from the ventral stream than in our daily life [25]. Secondly, more research is needed regarding the role of the dorsal stream in the specific sensorimotor deficits in ASD that would be targeted by an IVR paradigm in order to provide the best possible support for the improvement of sensorimotor skills. Indeed, one of the main goals in the field of IVR technologies is to achieve near-real-life binocular motion and depth perception [27,28]. 

Although IVR applications for people with ASD are growing for educational, entertainment, and treatment purposes, there is a lack of knowledge about how ASD sensorimotor atypicalities and individual variability might lead to different interactive processes and outcomes. Therefore, the present study presents a method that aims at shedding initial light on the differences between moving and perceiving in reality versus IVR for children and adults with ASD. The knowledge gained through this research will be fundamentally important in informing researchers and clinicians who are using this technology with this specific population. 

ASD presents a challenge for any individual involved in understanding, assessing, investigating, and treating those with the disorder. The wide variability of patient profiles requires us as researchers to struggle with methodology, embrace the uncertainty of complex phenomena, and be open, thoughtful, and modest in our research practice [29]. Given the contradictory evidence in the extant literature and the innovative aim of the present research, we adopted an exploratory, descriptive approach. As some statisticians have recently pointed out, “rather than focusing our study reports on uncertain conclusions, we should thus focus on describing accurately how the study was conducted, what problems occurred, what data were obtained” [30] (p. 262). Therefore, the aim of this pilot is to test the feasibility of the experimental procedure with children and adults with high- and low-functioning ASD, as well as to describe data characteristics. We will highlight the importance of exploring inter- and intra-individual differences, which contain meaningful information for assessment and intervention purposes. 

In sum, the aim of the present study is to investigate the extent to which the reliability of visual and proprioceptive information aids the self-motion accuracy of children and adults with ASD. To this end, we utilised a self-turn task and manipulated the way visuo-proprioceptive information was provided among unimodal and multimodal conditions. We also aim to explore whether HMD-delivered IVR, compared to equivalent real environments, affects self-motion accuracy, and to find whether the paradigm is feasible for use with this population.

## 2. Materials and Methods

*Participants.* For this pilot study, we recruited 4 male children (8−13 years old; M = 8.7; SD = 1.2) and 5 male adults (23−39 years old; M = 28.8; SD = 8.3) with a diagnosis of ASD confirmed by their clinicians (see Table 1 for demographic information). The experiment was explained to all parties and informed consent was obtained from parents and professionals responsible for each participant. The study was conducted in accordance with the Declaration of Helsinki, and the protocol was approved by the Ethics Committee of psychology research, University of Padova (Identification code 5A539475A80B5D451B7BC863210C8A61).

**Figure 1:**
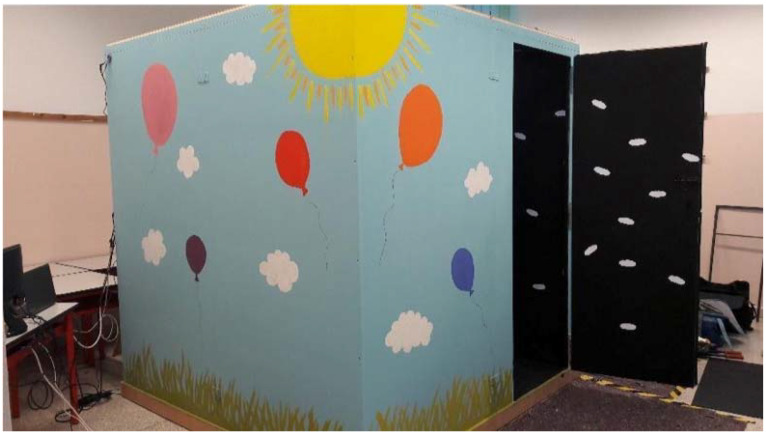
The testing room.

*Setup.* Materials and methods have been described in detail in our previous study with neurotypical children and adults [19]. The employed materials included a soundproof, 2x3 metre testing room with black interior walls where small white clouds were randomly fixed (see Figure 1), illumination, audio communication, and videotaping systems, and the HMD Oculus Gear VR 2016 (101° FOV, 345 g weight, 60 Hz refresh rate) interfaced with a Samsung Galaxy S7 (152 g weight) providing IVR simulations (360° pictures) of the testing room. 

*Procedure.* Participants were asked to sit on a swivel chair fixed in the centre of the testing room. For each trial, the experimenter manually rotated the chair a certain degree (passive rotation) from a *start position* to an *end position*. After each passive rotation, participants had to rotate back to the start position (active rotation). Participants’ stop position was recorded as the *return position*. The self-turn error was calculated in terms of degrees of absolute difference between the *start position* and the *return position*. Therefore, lower levels of error indicate higher accuracy.

Start, end, and return position data were manually coded by two independent raters of the video recordings. Inter-rater reliability was assessed via intra-class correlation (ICC). The intra-class correlation index (ICC) estimates an ICC = 1, with a 95% confidence interval being 1 < ICC < 1. This nearly perfect inter-coder agreement derives from the small mean difference between the two coders’ values within the huge range of possible values (0–360). The mean difference between coder A and coder B is minimal (M_A-B_ = 0.5).

*Experimental design and conditions.* In a within-subjects multifactorial (2 × 3) design, all participants were randomly exposed to two trials for each of six conditions (a small number of trials was used to keep the experiment as short as possible for participant comfort). The self-turn task was performed in two Environment conditions (Reality and IVR) for each of three Perception conditions (Only Proprioception, Only Vision, Vision + Proprioception). The IVR conditions involved wearing an HMD that showed 360° pictures of perceptually equivalent versions of the reality (R) conditions. The Only-Proprioception (P) condition removed all visual information (with a darkened room or HMD providing no input). The Only-Vision (V) condition limited the access to proprioceptively informative visual landmarks (hiding the participants’ body and the room corners) in order to disrupt proprioception, while providing a proprioceptively uninformative visual texture (a pattern of small bright clouds on the walls). The intention was to disrupt proprioception via an alteration of the visual information available without making changes to the proprioceptive information arising from participants’ bodies during the passive and active movements. Indeed, previous research has suggested that after being disorientated by a passive rotation in a real environment, people can still detect the position of global landmarks (the room’s corners), although they were found to make huge errors in locating surrounding objects [31]. The Vision + Proprioception (VP) condition allowed the participant to access reliable visual and proprioceptive information.

In order to diversify the passive rotations, they were executed both in clockwise and counterclockwise directions, with different amplitudes. Listed below are detailed descriptions of the six experimental conditions.

1. R_P (Reality; only proprioception: no visual information available; the room was completely darkened with no light source available).
2. R_V (Reality; only vision: proprioceptively uninformative visual texture of small bright clouds on the walls. No first-person view of the body or room corners in order to disrupt proprioception by manipulating vision).
3. R_VP (Reality; proprioceptively informative visual cues available, including first-person view of the body and room corners. The visual texture of clouds on the walls is available).
4. IVR_P (HMD on; only proprioception: no visual information available; HMD was worn with no visual input).
5. IVR_V (HMD on; only vision: proprioceptively uninformative visual texture of small bright clouds on the walls. No first-person view of the body or room corners in order to disrupt proprioception by manipulating vision).
6. IVR_VP (HMD on; proprioceptively informative visual cues available, including visible room corners, although the first-person view of the body is not visible. The visual texture of clouds on the walls is available).

All the analyses and graphical visualisations were conducted using the software R (version 3.6.1). The data were described through descriptive statistics and graphical representations, and results were interpreted from an exploratory perspective.

## 3. Results

The first aim of this pilot is to evaluate the feasibility of the experimental procedure with children and adults, even where severe conditions are present. One of the children (“C3”, 10 years old) enjoyed the swivel chair and played with it, rotating himself without complying with any verbal instruction provided. Another child (“C4”, 13 years old) disliked the testing room and refused to enter it to become familiar with the environment. Data from those participants could not be collected, and the descriptive analyses therefore include seven participants.

The seven participants included here demonstrated that they understood the instructions and task after a short training period. All participants readily wore the HMD. Among them, the two children required several breaks and verbal praise for remaining focused on the task. One of them (“C1”) was initially scared by the closing of the room door and by conditions performed in darkness, although he did decide to continue with the experiment. The other (“C2”) found the task boring and needed to be continuously motivated. One adult (“A4”) performed only the R_P condition and then exited the room, stopping the experiment. Due to technical issues, another adult (“A1”) performed the R_VP condition twice and did not perform the IVR_VP condition. The final dataset consisted of 24 observations from children and 50 observations from adults.

**Figure 2:**
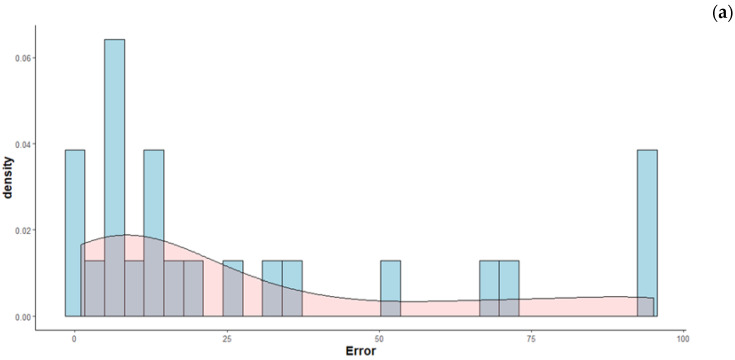

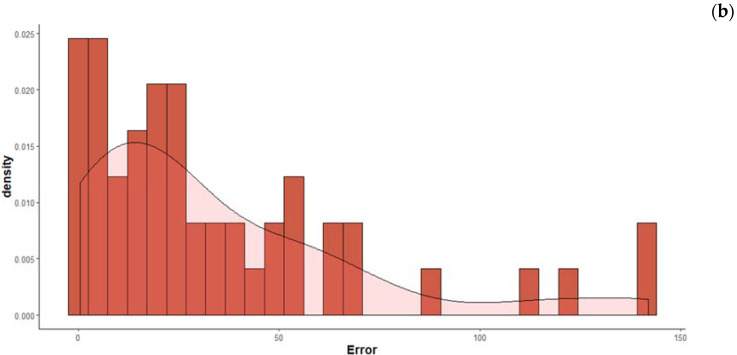
(**a**) Distributions of the observed self-turn error. Children (*n_participants_* = 2; *n_observations_* = 24). (**b**) Distributions of the observed self-turn error. Adults (*n_participants_* = 5; *n_observations_* = 50).

The mean self-turn error in the children’s sample was 28.4 degrees (SD = 32.3), while in the adults’ sample, it was 34.3 degrees (SD = 35.6). The distributions of the observed values have positive skewness, as visualised in Figure 2a,b.

**Figure 3:**
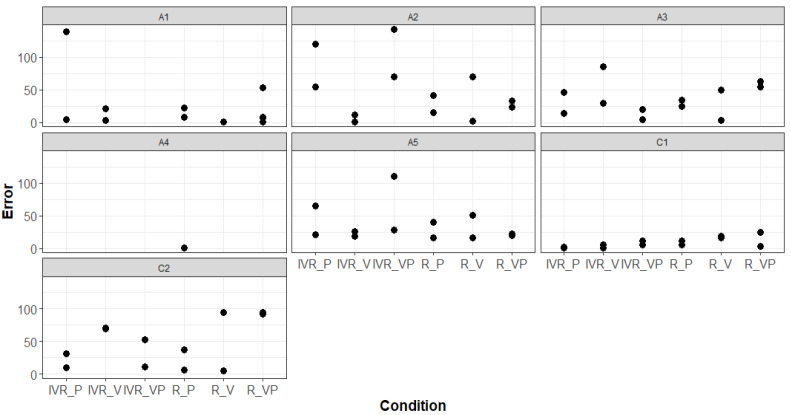
Self-turn error of single observations collected by each participant among conditions (*n_participants_* = 7; *n_observations_* = 74).

Exploring the main effect of experimental conditions, it is informative to look at individual observations, where we can appreciate that there is heterogeneity of performance (Figure 3).

Means and standard deviations of self-turn error according to age group and the experimental condition are reported in Table 2.

**Figure 4:**
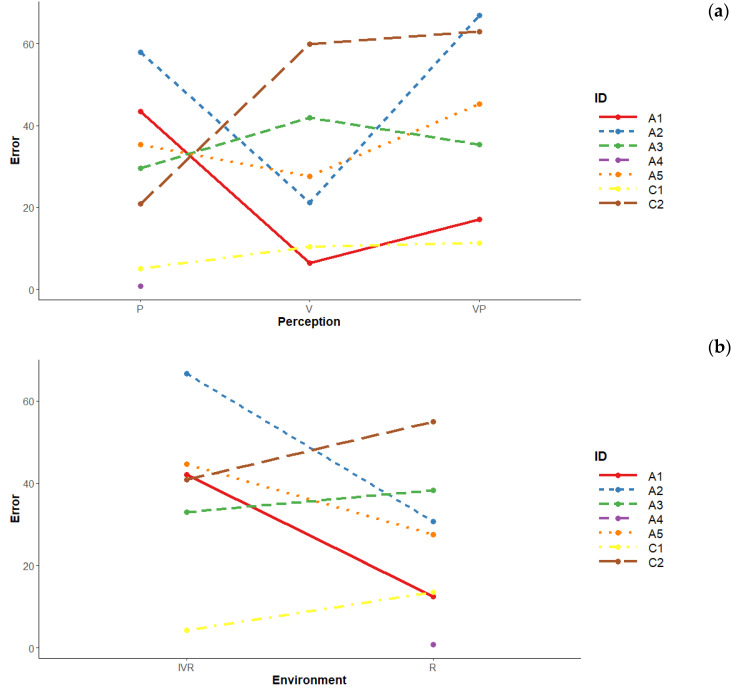
(**a**) Mean error made by each participant according to perception (marginalised over the other variables). (**b**) Mean error made by each participant according to environment (marginalised over the other variables).

Looking at the marginal role of perception and environment factors, we notice that those participants who perform worse in Only-Vision conditions and better in Only-Proprioception conditions seem to benefit from IVR (“A3”; “C1”; “C2”). Those who perform better with Only-Vision and worse with Only-Proprioception seem to be facilitated in Reality (“A1”; “A2”; “A5”) (Figure 4a,b).

**Figure 5:**
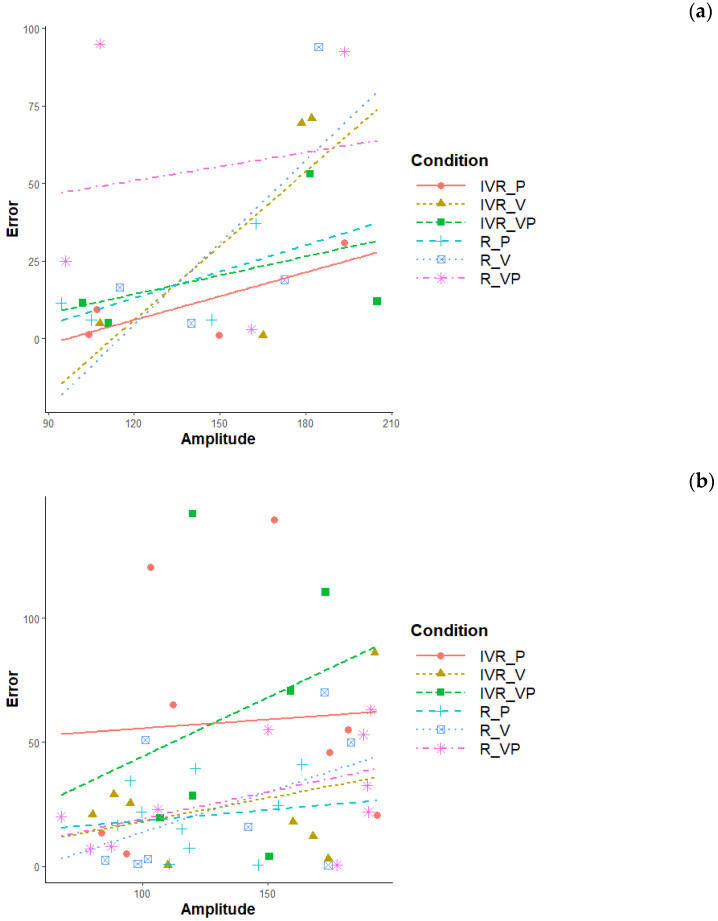
(**a**) Regression lines of self-turn error according to rotation amplitude in each condition. Children (*n_participants_* = 2; *n_observations_* = 24). (**b**) Regression lines of self-turn error according to rotation amplitude in each condition. Adults (*n_participants_* = 5; *n_observations_* = 50).

Trials were equally distributed among the two possible directions (N = 37 trials in clockwise and counterclockwise directions), which do not appear to affect the self-turn error (M_clockwise_ = 32.5; SD_clockwise_ = 34.3; M_counterclockwise_ 32.3; SD_counterclockwise_ = 35.1). The amplitude of passive rotations ranges from 67.5 to 205 degrees (M = 137.2; SD = 38.5). Although the effects of amplitude are not of main interest for this study, consistently with our previous findings [19], this variable is positively correlated with self-turn error. This association seems to be qualitatively different among conditions and age groups (Figure 5a,b). Increasing amplitude appeared to reduce children’s accuracy to the greatest extent in Only-Vision conditions performed in both Reality and IVR, while it reduced adults’ accuracy to the greatest extent in the Vision + Proprioception condition performed in IVR. Further investigation could specifically address this topic.

## 4. Discussion

This pilot study offers important initial insights regarding IVR research into the use of vision and proprioception in adults and children with ASD. The first finding with respect to feasibility is that all participants, including lower-functioning ones, readily accepted the use of HMD. Therefore, this appears to be a promising tool for research and treatment purposes in the field of severe ASD conditions, which are commonly understudied [32,33]. However, our experimental procedure requires participants to face some obstacles even when they understand the task and perform at a high level of accuracy. In this pilot study, we found that performance tended to fluctuate between within-condition trials and as such, averaging scores would make it difficult to detect an individual’s best performance due to interfering factors such as emotional state, motivation, skills of behavioural management, and fluctuations in attention. Future research could adapt the experiment to build a more engaging, game-like activity and include frequent rewards for participation to create a more attractive testing environment for participants. Moreover, a detailed evaluation of within-participant outlying performances could be run to detect the best performance the individual can show, rather than an average, which obscures these nuances.

As we only present preliminary data from a small sample, we make no inferential claims here. However, we do find this data informative for modest and cautious considerations. First of all, this methodology could show individual differences in the sensory conditions that facilitate self-motion. Moreover, we could distinguish between the individuals that may benefit more or be more impaired by using HMDs. Within the present sample, those who were facilitated by moving when proprioception was available and no vision was present also benefited from IVR. We cannot generalise this result to the whole population of individuals with ASD, but we strongly suggest that researchers and clinicians keep in mind that this technology can either facilitate or impair individuals depending on their profiles. For instance, an IVR training could be particularly effective for individuals who have reduced reliance on vision in reality. We can speculate that the limited use of external stimuli to calibrate internal body-based information might lead to early motor impairments and therefore stereotypy, which refers to restricted repetitive behaviours and interests which reduce the individuals’ learning opportunities and interfere with development [34]. Therefore, future research on the potential of IVR training could select people with reduced use of vision for paradigms aimed at learning within IVR and assess outcomes such as improvements in sensorimotor functions, reduction of stereotypies, and cascading benefits on higher-order cognitive and socio-communicative abilities.

Finally, the present pilot study has some limitations, which call for future research using this promising paradigm. The first limitation is that the experimenter manually rotated the participant, and as such, although experimenters were trained to keep a similar speed and method of rotating, the rotation velocity was not perfectly consistent across trials and participants, which could potentially have influenced participants’ performance. The second main limitation was the small sample size, which we plan to enlarge in future studies. This would allow us to explore the effect of other relevant factors such as age, comorbidities, and level of general functioning on individual variability. To this end, we aim to extend our measurements and assess other symptoms that could be associated with visuo-proprioceptive atypicalities, such as sensory profile, fine and gross motor abilities, severity of stereotypies and repetitive behaviours, and communicative and social skills.

The method presented here has been previously investigated with neurotypical children and adults [19]. Bayesian model comparison analyses suggested that the sensory information available and the type of environment might result in a perception x environment interaction effect. Therefore, the role of visuo-proprioceptive information might be different in the two environments. Future studies with individuals with ASD could investigate this interaction effect to explore whether different sensory strategies facilitate self-motion in either reality or IVR. Moreover, in a paper in preparation [35], we have further investigated the memory effect of the rotation amplitude (namely, the amount of information to be encoded and reproduced) of our self-turn paradigm, with findings suggesting that the encoding of own body location is facilitated when vision and proprioception are optimally integrated. Consistent with those findings, the present pilot indicates that rotation amplitude might differently affect accuracy across conditions. Our future research with people with ASD could expand on which experimental conditions are most disrupted by memory load.

There is a long way to go, and the present study is just a first indication. As of March 2020, when searching for “Vision” AND “Proprioception” AND “Autism”, Scopus provides only 25 documents. Following the first experimental study published in 1983 [36], there was a gap until 2005 for the next theoretical one [37]. Further experimental research is needed to shed light on this early domain-general sensorimotor mechanism that potentially has huge implications for development.

## 5. Conclusions

The present pilot study offers preliminary insights into how the self-motion accuracy of children and adults with ASD is affected by individual differences in the way they rely on vision and proprioception, and in how they interact with real environments and IVR. Preliminary results suggest that inter-individual variability in sensorimotor functioning has a meaningful impact on the possibility for people with the heterogeneous conditions of ASD to be facilitated by perceiving, moving, and therefore learning in IVR. Importantly, this research also found this paradigm and the use of an HMD to be acceptable and feasible with the present sample, indicating good potential for future research utilising these methods.

## Figures and Tables

**Table 1:** Participants’ demographic information.

Participant	Age	Diagnosis
C1	8	ASD, ADHD ^1^, ODD ^2^, Dysgraphia
C2	8	ASD, Mild ID ^3^
C3	10	ASD, Mild ID
C4	13	ASD, Moderate ID
A1	36	ASD, Severe ID
A2	26	ASD, Mild ID
A3	20	ASD, Mild ID
A4	23	ASD, Mild ID
A5	39	ASD, Severe ID

^1^ ADHD (Attention Deficit Hyperactivity Disorder); ^2^ ODD (Oppositional Defiant Disorder); ^3^ ID (Intellectual Disability).

**Table 2:** Means and standard deviations of self-turn error according to age group and the experimental condition.

Age Group	Condition					
	R_P	R_V	R_VP	IVR_P	IVR_V	IVR_VP
**Children**	15.1 (14.8)	33.6 (40.7)	53.9 (47)	10.8 (14.1)	36.6 (38.9)	20.4 (22)
**Adults**	20.2 (14.9)	24.3 (28.2)	28.4 (21.9)	58.1 (49.2)	24.4 (26.9)	62.5 (55)

Note: Standard deviations are reported in brackets. (*n_participants_* = 7; *n_observations_* = 74).
